# Comparative Evaluation of Major Robotic Systems in Microanastomosis Procedures: A Systematic Review of Current Capabilities and Future Potential

**DOI:** 10.3390/brainsci14121235

**Published:** 2024-12-09

**Authors:** Delia Cannizzaro, Matteo Scalise, Chiara Zancanella, Stefano Paulli, Stefano Peron, Roberto Stefini

**Affiliations:** 1 Department of Neurosurgery, ASST Ovest Milano Legnano Hospital, 20025 Legnano, Italy; 2 Vita-Salute San Raffaele University, School of Medicine, Via Olgettina 58, 20132 Milan, Italy; 3 Department of Molecular Medicine, University of Pavia, Via Forlanini 14, 27100 Pavia, Italy; chiara.zancanella01@universitadipavia.it; 4 Department of Maxillofacial Surgery, ASST Ovest Milano Legnano Hospital, 20025 Legnano, Italy; stefano.paulli@asst-ovestmi.it

**Keywords:** robotic-assisted surgery, microsurgery, robotic systems, neurosurgery, vascular anastomosis

## Abstract

**Background/Objectives:** Robotic-assisted surgery has revolutionised modern medicine, enabling greater precision and control, particularly in microsurgical procedures. This systematic review evaluates the current state of robotic-assisted surgery across various specialties, focusing on four major robotic systems: Symani, Da Vinci, ZEUS, and MUSA. **Methods:** The review systematically analyses the effectiveness of these systems in performing vascular, lymphatic, and nervous anastomoses, comparing key metrics such as procedure time, success rates, and learning curves against manual techniques. It includes 48 studies, highlighting the technological capabilities and limitations of these systems in direct comparisons. **Results:** Results indicate that while robotic procedures often take longer than manual methods, significant improvements in efficiency are observed as surgeons gain experience. **Conclusions:** Overall, this study provides insights into the future potential of robotic-assisted surgery and highlights areas that require further research. It ultimately aims to promote the application of robotic systems in cranial neurosurgery, with a particular focus on advancing neurovascular techniques, such as microsuturing for bypass procedures.

## 1. Introduction

Robotic-assisted surgery represents one of the most significant technological advancements in modern medicine, offering enhanced precision, control, and potential for minimally invasive procedures [1]. Over the past two decades, the use of robotic systems in surgery has expanded across various medical specialties, particularly in complex microsurgical tasks [2,3]. However, the adoption of this technology has also raised critical questions about its efficiency, learning curve, and comparative outcomes when evaluated against traditional manual techniques [4,5]. This manuscript systematically reviews the current landscape of robotic-assisted surgery, with a focus on its application in different surgical fields such as plastic and reconstructive surgery, maxillofacial surgery, and cardiac surgery. By analysing four primary robotic systems—Symani, Da Vinci, ZEUS, and MUSA—we aim to assess their technological capabilities, usage patterns, and effectiveness in procedures such as vascular and nervous anastomoses. The review examines key performance metrics such as procedure time, success rates, and surgeon experience while identifying limitations in the existing literature that hinder direct comparisons between robotic and manual methods. Overall, this study seeks to provide a comprehensive evaluation of the current state of robotic-assisted surgery, offering insights into its future potential and areas for improvement. Building on this, the ultimate purpose is to promote the adoption of robotic systems in cranial neurosurgery, with a specific emphasis on improving neurovascular procedures, such as microsuturing in bypass surgery.

## 2. Materials and Methods

This systematic review was conducted according to Preferred Reporting Items for Systematic Reviews and Meta-Analyses (PRISMA) [6]. The review was not registered.

### 2.1. Search Strategy

A systematic search was performed in three different databases (PubMed/Medline, Web of Science (WoS), Scopus) up to the 17th of September 2024.

On PubMed/Medline, MESH terms and keywords were incorporated in the search strategy, such as: “symani” OR “symani robotic system” OR “neuroarm” OR “neuroarm robotic system” OR “musa” OR “musa robotic system” OR “zeus” OR “zeus robotic system” OR “da vinci” OR “da vinci robotic system” AND “microsurgery”.

The corresponding search strategy was applied for Web of Science and Scopus. The cited robot systems were chosen as they were considered the most suitable for surgical microanastomoses.

Four different robotic surgical systems were used in different settings and studies: the Symani Surgical System, the Da Vinci Surgical System, the ZEUS Robotic Surgical System (ZRSS), and the MUSA Surgical System. The Symani and Da Vinci systems are the most featured, with *n* = 20 and *n* = 19 articles, respectively, followed by the ZRSS (*n* = 5) and MUSA (*n* = 4).

No language filter was initially applied to the search. No date filters were applied.

### 2.2. Eligibility Criteria

The selected studies met the following inclusion criteria: (1) written in English; (2) included robot-assisted procedures on vascular, lymphatic, or nerve anastomoses; (3) included either in vivo (human or animal), cadaveric, ex vivo, or synthetic models; and (4) described technical data or outcomes of the anastomoses.

Except for reviews or meta-analyses, all study designs were considered.

Two reviewers (M.S. and C.Z.) independently assessed articles for inclusion, extracted data, and reviewed the methodological quality. Discrepancies were resolved by a senior author (D.C.).

### 2.3. Data Extraction

The data were entered into a customised Microsoft Excel^®^ (Version 16.89) spreadsheet. Data included studies’ characteristics (authors, discipline of interest, journal, year of publication); methodological details (study design, type of robotic surgical system used, type of surgical model used, type and number of anastomoses); technical information on the anastomosis performed (anastomosis time, graft calibre, number of stitches per anastomosis, suture dimension); and anastomoses’ outcome measurements.

When a comparison group involving manual (non-robotic-assisted) anastomosis procedures was present, relevant data were extracted, and a comparative analysis between the two groups was conducted.

### 2.4. Risk of Bias Assessment

The study underwent a detailed evaluation for potential sources of bias that might influence the validity of its findings. Two reviewers (M.S. and C.Z.) analysed the articles, with each article being reviewed independently. Any discrepancies were resolved with input from a senior reviewer (D.C.). No specific tools were employed to evaluate the methodological quality of the studies. While errors were identified in each study analysed, they could not be rectified, and the combined risks arising from individual data extractions remained unresolved.

## 3. Results

### 3.1. Selection of Studies

The search produced 467 articles (PubMed *n* = 122, WoS *n* = 130, Scopus *n* = 215).

Duplicates identified from the analysed databases (*n* = 197) were removed to ensure a non-redundant dataset for subsequent analysis.

Non-English written articles (*n* = 21) were removed according to the inclusion criteria.

Overall, 249 records underwent a first screening, and 194 were excluded based on titles or abstracts not meeting the criteria.

Overall, full-text review was performed on 55 articles, with 7 being excluded for either wrong study design (as systematic reviews) (*n* = 4), wrong intervention (*n* = 1), wrong population (*n* = 1), or absence of suitable data (*n* = 1).

Ultimately, the inclusion criteria were fulfilled by 48 articles, which were included in the systematic review.

**Figure 1:**
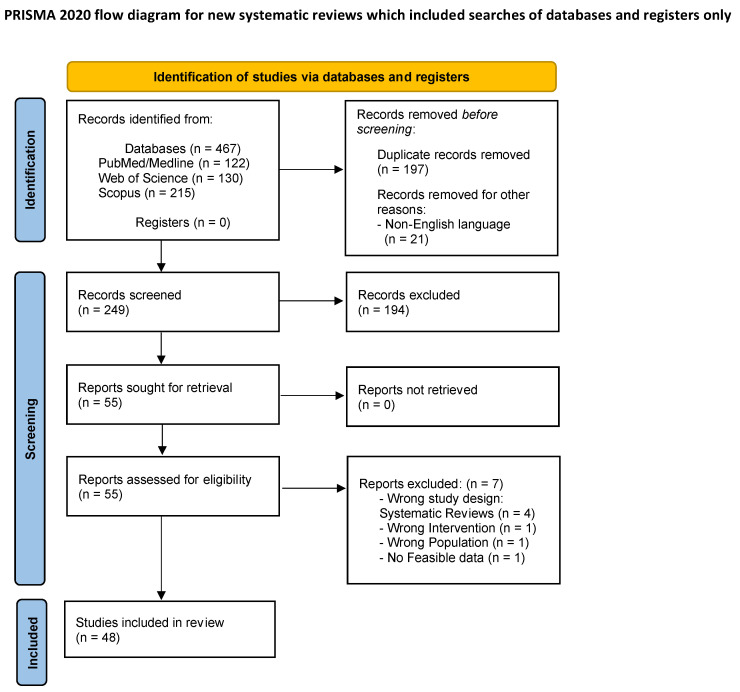
PRISMA 2020 flow diagram for new systematic reviews.

Figure 1 shows the PRISMA 2020 flow diagram for systematic reviews with the selection process of studies identified and included.

### 3.2. Studies’ Characteristics

Data from the 48 articles were extracted, and the characteristics of the studies were analysed.

Table 1 contains all the information on the characteristics of the studies, including study design, discipline of interest, robotic surgical system, and presence/absence of a comparative non-robot-assisted group.

### 3.3. Design of the Studies

Among the 48 studies analysed, 17 are case series, 8 are case reports, 20 are preclinical studies, 2 are randomised controlled trials, and 1 is a non-randomised clinical trial.

Another aspect that deserves attention is the field of interest associated with the single articles. “Plastic and Reconstructive Surgery” is by far the most represented, with n = 33 articles; succeeded by “Maxillofacial Surgery” (*n* = 4), “Cardiac Surgery” (*n* = 3), “Neurosurgery” (*n* = 4), “Thoracic Surgery” (*n* = 1), “Orthopedic Surgery” (*n* = 1), “Vascular Surgery” (*n* = 1) and “Urology” (*n* = 1).

### 3.4. Surgical Models

Surgical models were categorised into four subgroups: “in vivo models”, comprehending either human or animal subjects (n = 508); “cadaveric human models”(*n* = 3); “ex vivo models” (*n* = 51); and “synthetic models” (*n* = 276).

Three articles [41,46,49] did not specify the number of subjects, only addressing the type of model used; accordingly, the subsequent analysis did not consider these articles for subject data calculations.

The articles considered in this analysis overall include 838 models.

Most of the samples were in vivo models with a total of 508, comprising 365 human patients and 143 animals (134 rats, 5 pigs, 2 mice, 2 dogs), which accounted for 60.62% of the total models. Additionally, there were 3 human cadaveric models (0.38%) and 51 ex vivo models, including 20 porcine specimens (18 hearts, 2 n.s.), 30 earthworm specimens, and 1 specimen from rats, making up 6.08%. Finally, 276 synthetic vessels were used, with 126 made of silicone and 150 from synthetic polyvinyl alcohol, representing 32.93% of the total models.

### 3.5. Anastomoses’ Type and Number

Anastomoses were categorised into three different subgroups: “vascular”, “lymphatic”, and “nervous”. A total of 1153 anastomoses were taken into consideration, with vascular anastomoses being the most prevalent (*n* = 903), followed by nerve anastomoses (*n* = 120) and lymphatic (*n* = 115).

One study [47] described a fourth kind of anastomosis, using earthworm bodies as samples, which were counted under “other types of anastomoses” (*n* = 15), but, due to the small size of the sample, it did not affect the following analysis.

Two articles [36,51] did not specify the number of anastomoses performed and thus were not included in the results.

Vascular anastomoses account for 78.32% of the total, nervous for 10.40%, and lymphatic for 9.97%.

Among vascular anastomoses, 454 were arterial (50.28%), 161 were venous (17.83%), 6 were arterio-venous (0.66%), 6 were not specified (0.66%), and 276 were synthetic vessels (30.56%).

As per the lymphatic anastomoses group, lymphatic-venous anastomoses (LVAs) accounted for 95.65%, leaving the lympho-lymphatic anastomoses (LLAs) as the remaining 4.35%.

When specified, the way anastomoses were performed was also considered; 380 were executed in an end-to-end manner (82.60%) and 80 in an end-to-side manner (17.40%).

### 3.6. Anastomosis Time

Referring to robot-assisted procedures, the mean time of suturing was analysed separately for arterial, venous, lymphatic, nerve, and synthetic anastomoses.

When a comparison group was provided, these data were compared to the suturing times achieved with manual microsurgical techniques.

Nineteen articles (38%) did not assess the anastomosis time.

#### 3.6.1. Arterial Anastomoses

The mean time for a single robot-assisted arterial anastomosis was 29.16 min.

Five articles assessed the comparison between robotic and manual anastomosis time for arterial grafts; Table 2 reports the comparison.

Analysing their data together, the mean time for arterial anastomosis was 20.73 min, while the mean time for manual anastomosis was 11.97 min, with a mean difference between the robotic and manual time of 8.75 min.

Barbon et al., 2022 [14] did not differentiate arterial and lymphatic anastomoses in the counting of anastomosis time, referring to a mean time of 25.3 ± 12.3 min for robotic and 14.1 ± 4.3 min for manual ones (*p* < 0.01).

Lai et al., 2019 [26], did not differentiate between arterial and venous anastomoses in the counting of anastomosis time, referring to a mean time of 38.4 ± 10.4 min for robotic and 28.0 ± 7.7 min for manual ones (*p* < 0.001).

#### 3.6.2. Venous Anastomoses

All studies considered that the mean time for a single robot-assisted venous anastomosis was 35.04 min.

Three articles assessed the comparison between robotic and manual anastomosis time for venous grafts; Table 3 describes these data.

Analysing the data of the three articles together, the mean time for venous anastomosis was 24.25 min, while the mean time for manual anastomosis was 12.27 min, with a mean difference between the robotic and manual time of 11.98 min.

#### 3.6.3. Lymphatic Anastomoses

Regarding lymphatic anastomoses, the mean suturing time was 27.82 min.

Only one article [8] assessed a comparison between robotic and manual anastomosis time for lymphatic grafts: robotic 25 ± 6 min, manual 9 ± 6 min (*p* < 0.001).

#### 3.6.4. Nervous Anastomoses

The mean time of nervous anastomoses was 37.92 min.

#### 3.6.5. Synthetic Anastomoses

Synthetic anastomoses’ mean time was 19.36 min. Wessel et al., 2024 [19], reported a direct comparison between robotic (24 min) and manual (13.5 min) times using synthetic grafts.

Two of the analysed articles [28,47] did not report specific times for robotic and manual anastomosis but simply outlined a time difference between the two methods.

Overall, in 83.33% of cases (*n* = 10), the manual method performed faster than the robot-assisted one. In the remaining 16.67% of cases (*n* = 2 [13,28]), there was no statistically relevant difference in time between the two groups.

Notably, in the majority of cases, anastomoses were performed by experts who had little or no training in robotic surgery. Conversely, many reported a positive learning curve, which improved anastomoses time as the procedure was repeated.

#### 3.6.6. Robot Comparison of Anastomosis Time

Out of the 29 articles reporting the anastomosis time, 24.1% (*n* = 7) used the Da Vinci Surgical System, 6% (*n* = 2) used MUSA models, 55.17% used the Symani Surgical System, and 10.3% used the Zeus robotic Surgical System. Comparison of anastomosis times between robots is challenging due to the difference in the number of articles and the type of suture performed.

Overall, the Symani Surgical System was better described in terms of suturing time, as it was the only system with data on all types of sutures, and it demonstrated what appears to be the fastest overall performance time.

On the other hand, the Da Vinci system’s performance time was assessed only for vascular and nervous anastomoses, Zeus only for arterial, and MUSA only for lymphatic.

### 3.7. Graft Caliber, Number of Stitches per Anastomosis, and Suture Dimension

On average, the graft calibre used for robotic-assisted anastomosis was around 1.82 mm. Notably, 27 articles (55%) did not specify the calibre used.

Arterial vessels had an average calibre of 1.50 mm, and veins had an average of 1.49 mm. Lymphatic grafts had an average calibre of 0.63 mm, while nerves had an average of 2.65 mm. Synthetic anastomoses used models with an average calibre of 1.33 mm. One article [47] used an animal earthworm specimen to simulate a graft, and the average calibre was 2 mm.

According to the robotic system used, different anastomotic calibres were reported: the Da Vinci Surgical System was utilised to perform anastomoses on grafts as small as 1.3 mm in diameter (venous grafts) or 1.5 mm (arterial) [39]. MUSA systems were associated with grafts as small as 0.38 mm (venous) or 0.49 mm (lymphatic) [18]. The Symani Surgical System was used in anastomoses with grafts as small as 0.6 mm (arterial) [23], 0.7 mm (venous) [21], and 0.4 mm (lymphatic) [11]. The Zeus Robotic Surgical System was used on 1 mm arterial vessels [49]. Due to the paucity of data on graft calibres, further comparison between robotic systems could not be performed. Nevertheless, these data attest to the feasibility of robotic surgical systems in performing anastomoses on vessels and grafts with calibres even smaller than 1 mm.

The mean number of stitches per anastomosis was 7.212. However, 27 articles (55%) did not report these data.

Different types of suture threads were used, ranging from 4-0 to 12-0. More specifically, 4-0, 5-0, and 6-0 sutures were used once; 7-0 was used 4 times; 8-0 was used 8 times; 9-0 was used 11 times; 10-0 was used 18 times; 11-0 was used 6 times; and 12-0 was used 2 times.

### 3.8. Anastomoses Outcome Measurements

#### 3.8.1. Anastomosis Patency

Across the articles analysed, 31 of them reported the patency rate of the anastomoses performed at T0, with an overall mean patency of 97.36%.

Other papers reported patency rates at different follow-up times:

Menichini et al., 2024 [13], assessed a patency rate of 100% at 28 days after the procedure.

Damiano et al., 2001 [54], assessed a 93% patency rate at 2 months of follow-up.

Malzone et al., 2023 [17], assessed the patency rate of robotic arterial anastomoses at 1 week and venous anastomoses 2 weeks after the procedure, reporting a 91% and 91% patency, respectively. Moreover, the article compared these results with those of the manual anastomoses group: Arterial anastomoses had a 100% patency rate after one week of follow-up; the venous anastomoses had an 85% patency rate after 2 weeks of follow-up. However, differences between robotic and manual groups were not statistically significant (*p* > 0.05).

Van Mulken et al., 2020 [8], and van Mulken et al., 2022 [9], assessed the patency of the same cohort of anastomoses at two different time points: at T0 [8] and at the 1-year follow-up [9]. Initially, robotic-assisted anastomoses had a 100% patency rate. At one year of follow-up, 66.6% of patients had at least one patent anastomosis compared to 81.8% in the manual anastomoses group.

The studies analysed performed different tests to assess the patency rate. These included Acland’s milking test, indocyanine green (ICG), the patent blue test, the NaCl test, and post-operative MR lymphangiography.

#### 3.8.2. Complete Flap Loss

Only 3 articles reported complete flap loss, with Tolksdorf et al., 2024, reporting *n* = 2 flap loss cases on *n* = 59 total anastomoses (3.38%) [22], Beier et al., 2023, *n* = 1 flap loss out of 23 (4.34%) [29], and Gorji et al., 2024, *n* = 1 out of 23 anastomoses (4.34%) [31].

#### 3.8.3. Partial Flap Loss

A total of 3 cases of partial flap loss were reported: Mori et al., 2024, reported *n* = 1 partial flap loss out of the 40 anastomoses (2.5%) [10], Struebing et al., 2024, *n* = 1 out of the 96 anastomoses (1.04%) [11], and Gorji et al., 2024, reported one case of less than 5% loss of total flap surface area [31].

#### 3.8.4. Need for Revision or Conversion to Manual Technique

Out of the 1153 anastomoses, only 18 needed revision, which accounted for 1.6% of cases. No difference in the need for revision was outlined between robotic and manual anastomosis.

Conversion to the traditional anastomosis technique was described as necessary in five cases, accounting for 0.43% of the total number of anastomoses considered in this analysis.

### 3.9. Learning Curve

Fifteen articles (30.61%) reported a positive trend in the learning curve of users for robotic-assisted suturing techniques. Referring to anastomosis time reduction, as the robotic systems were utilised more, the anastomoses were performed faster, with reported improved manual dexterity and smaller error rates. Table 4 contains all the available data on the improvement rate in anastomosis time.

Thirteen articles detailed a significant improvement as the robotic system was used: across all studies, an average improvement of 41.8% in anastomotic time was observed.

## 4. Discussion

This systematic review of research on robotic-assisted surgical systems for microanastomoses across various medical specialties offers important insights into the advancement of surgical innovation. It is important to note that most excluded studies did not conform to the required study design, population, or type of intervention. This stringent selection process guaranteed that the articles included were directly relevant to the research question. The prevalence of studies in plastic and reconstructive surgery, as compared to other surgical fields such as maxillofacial surgery and cardiac surgery, facilitated a concentrated analysis on microanastomoses. This trend highlights the intricate precision demanded in plastic surgery, where robotic assistance may provide considerable benefits in reducing human error and enhancing surgical outcomes.

The four robotic surgical systems examined—Symani, Da Vinci, ZEUS, and MUSA—illustrate the variety of technologies currently available. The Symani [11] and Da Vinci [26] systems, with 20 and 19 studies, respectively, dominate the existing literature, indicating their broader acceptance and potentially more advanced development compared to ZEUS [54] and MUSA [8]. These systems possess differing technical capabilities, with Symani excelling in microsurgery, while Da Vinci is more widely utilised across diverse surgical domains. The limited number of studies on ZEUS and MUSA may be due to their more specialised or emerging applications.

The employment of diverse surgical models (in vivo, cadaveric, ex vivo, and synthetic) underscores the extensive experimentation and validation necessary for emerging surgical technologies. A notable 60.62% of studies utilised in vivo models, with human patients constituting the largest subset. The considerable representation of synthetic models highlights the critical role of artificial simulations in evaluating and enhancing robotic systems prior to their clinical deployment. Notably, the use of earthworm specimens as ex vivo models indicates creative methodologies for investigating vascular anastomoses, although their biological relevance to human surgery may be somewhat constrained.

Vascular anastomoses were the most commonly studied, followed by nervous and lymphatic anastomoses. This trend highlights the significance of accurate and consistent suturing in cardiovascular and microvascular procedures. Nevertheless, the duration of robot-assisted anastomoses remains a significant concern. On average, robotic arterial anastomoses required 29.16 min, which is considerably longer than the 11.97 min typically needed for manual procedures. This time discrepancy of 8.75 min raises questions about the practical efficiency of robotic systems; however, the enhanced precision provided by robotic assistance may justify the extra time, especially in more complex cases.

The duration of venous anastomoses in robot-assisted procedures was similarly prolonged, with an average time of 35.04 min compared to manual techniques. Although robotic systems are anticipated to decrease human error and enhance outcomes, the longer procedural time continues to pose a challenge. Furthermore, the variability among the studies, with differing protocols for documenting anastomosis times, makes it difficult to directly compare these results.

The findings from the three studies on venous anastomoses [17,22,34] indicate a considerable mean time disparity between robotic (24.25 min) and manual (12.27 min) procedures, with an 11.98-min advantage for manual techniques. However, the limited sample size presents a significant drawback, hindering the ability to generalise these results to wider contexts. Although the time difference is considerable, a more extensive dataset would be essential to determine whether this disparity remains statistically significant across various surgeons and robotic systems.

The examination of lymphatic anastomoses, featuring just one article [8] that compared robotic (25 ± 6 min) and manual (9 ± 6 min) times, highlights the considerable learning curve and time commitment associated with robotic procedures. The notable time disparity indicates existing inefficiencies in robotic systems for intricate tasks such as lymphatic grafts. Likewise, the data for nervous anastomoses (mean 37.92 min) imply that these procedures are especially time-consuming, emphasising the necessity for continued enhancement of robotic platforms and techniques in these specific contexts.

Synthetic anastomoses exhibited a smaller time discrepancy, with robotic procedures averaging 19.36 min compared to 13.5 min for manual methods. While robotic techniques were generally slower in most instances, this category of anastomoses indicates that as robotic technology advances and surgeons gain experience, the time difference may diminish. Furthermore, it is promising that in 16.67% of cases, there was no statistically significant difference in performance times between robotic and manual methods, suggesting that robotic systems are becoming increasingly competitive in terms of efficiency.

The diversity of graft calibres utilised in robotic-assisted anastomoses, which averages approximately 1.82 mm, demonstrates the precision capabilities of these systems, especially when handling small-calibre vessels. However, the absence of data regarding graft calibre and the number of stitches per anastomosis in over half of the articles represents a notable limitation in the existing literature. Additionally, the use of various suture dimensions (spanning from 4-0 to 12-0) complicates comparisons, as different thread sizes can affect both the complexity of the procedure and the time taken for completion. Establishing standardised reporting of this data across studies is crucial for facilitating more meaningful comparisons in future research.

The high patency rates of anastomoses across studies highlight the effectiveness of both robotic and manual techniques in preserving vascular integrity immediately post surgery. However, follow-up data exhibit considerable variability. While some studies reported 100% patency rates at 28 days [13], others, such as Van Mulken et al. [9], indicated a drop to 66.6% at 1 year for robotic anastomoses. In comparison, the manual group achieved a patency rate of 81.8%, suggesting potential long-term challenges associated with robotic procedures that may not be evident in short-term evaluations. The limited data on long-term outcomes underscores the necessity for more comprehensive follow-up studies to evaluate the durability of robotic-assisted anastomoses.

One of the most encouraging discoveries is the notable reduction in anastomosis times as surgeons gained more experience with robotic systems. The learning curve was reported in 30.61% of the articles, indicating an average enhancement of 41.8% in anastomosis time. This implies that, although robotic procedures might initially require more time, surgeons can swiftly acclimate, leading to improvements in both efficiency and accuracy over time. The favourable trend observed in the learning curve reinforces the notion that greater adoption and training in robotic surgery could result in quicker, more dependable procedures in the future. Among the robotic systems, the Symani Surgical System demonstrated the fastest anastomosis times across various suture types, especially when compared to the Da Vinci, ZEUS, and MUSA systems. However, the absence of direct comparisons between these robots, along with differences in study designs and the types of anastomoses evaluated, complicates the overall analysis. Most studies centred on Symani for vascular and nerve anastomoses, while Da Vinci and ZEUS were primarily used for arterial procedures. These fragmented data pose challenges in drawing definitive conclusions about the relative performance of each system.

Despite the benefits provided by robotic-assisted procedures, including faster recovery and decreased hospital length of stay, robotic surgery entails high costs and specialised training.

Cost-effectiveness is influenced by case volume and societal perspective. Factors such as the length of stay or operative time are highly dependent on the operator and surgical team, and thus the feasibility of this technology is in strict relationship with the centre’s experience. Given the steep learning curve, robotic surgery has the potential to counterbalance, in the long term, the initial equipment and training costs [55]. Certainly, the economic feasibility of highly advanced technology in medicine is justified only when tailored to the specific field and operation. Currently, robotic surgery is mainly utilised in general surgery, urology, and gynaecology [56], although the analysed studies advocate for its potential in other fields as well. Further analysis is needed to compare the cost-benefit ratio in robotic and laparoscopic surgery [56]. Performing multiple anastomoses robotically might offset the relative costs in terms of the outcome’s benefits.

The usability and user adoption of robotic surgery are two key topics to address.

It takes considerable conviction to trial a new technology when established techniques already offer proven benefits for patients.

While robotic surgery undoubtedly presents many advantages in microsurgery, there are several challenges that even an experienced microsurgeon must adjust to.

Reilly et al., 2024 [18], analysed surgeons’ efforts and frustration following their first trial of robotic-assisted anastomosis using a postoperative workload survey (the Likert scale). The results revealed an average frustration score of 15/20, with a mean effort score of 14.6/20. However, reductions in surgeon-reported frustration and effort were both observed over time.

An integral part of our job, for the advancement of our specialties and the benefit of our patients, is to challenge established techniques with new techniques. However, this often comes at a cost to the surgeon in terms of time; the time spent learning a new technique, training nursing staff, preparing the operating room, and, most importantly, the patience and motivation needed to master a new technique. There is surely a considerable initial challenge, but it can be successfully overcome after completing a sufficient number of cases [18].

Notably, one point in favour of robotic surgery is linked to surgery-related safety, especially in microsurgical procedures where imprecise or unintentional hand movements can pose significant risks. Tolksdorf et al., 2024 [22], pointed out how the seven-to-twenty-fold motion scaling and the tremor filter facilitate operations on small vessels. These features are designed to minimise human error and amplify dexterity, which is particularly valuable in environments requiring extreme accuracy; they therefore represent promising features for future microsurgery.

A key focus of this study is advocating for the widespread adoption of robotic systems in cranial neurosurgery. As surgical techniques continue to evolve, the integration of robotic technology holds significant potential to improve the outcomes of complex procedures. In particular, the neurovascular field stands to benefit greatly from robotic assistance, especially in delicate tasks such as vascular anastomoses for revascularization. These procedures require exceptional dexterity and precision, both of which can be enhanced by robotic systems that offer greater stability and control compared to traditional manual techniques. 

### Limitations of the Study

A significant limitation of this review is the lack of uniformity in reporting anastomosis times and procedural details across studies. For instance, certain studies did not differentiate between arterial, venous, and lymphatic anastomoses, while others did not specify the number of anastomoses performed. Moreover, although we recognised that including preclinical and human cadaveric studies may affect the consistency of the analysis, we chose to incorporate them to offer a more comprehensive perspective and to address the limited availability of published data on robotic-assisted anastomosis. These inconsistencies limit the ability to draw definitive conclusions about the relative efficiency of robotic versus manual techniques.

## 5. Conclusions

The analysis of robotic-assisted anastomoses reveals both the promise and limitations of this technology compared to manual techniques. While robotic systems enhance precision and reduce human error, they also exhibit significant time disparities, raising concerns about their efficiency in clinical settings. However, as surgeons gain experience, they can improve their efficiency with robotic systems, potentially narrowing the time gap with manual methods. This emphasises the need for ongoing training and the broader adoption of robotic technology in surgery. Despite longer procedure times, the precision and consistency of robotic systems, particularly in complex microsurgical tasks, show potential for better patient outcomes. There is a need for more comprehensive follow-up studies to assess long-term results and the durability of robotic-assisted anastomoses, given the current variability in data.

Overall, while robotic-assisted surgical systems offer exciting possibilities for the future of surgery, further research is essential to overcome existing limitations and fully leverage their potential in clinical practice. Increased collaboration between researchers and practitioners can drive innovation, enhance training protocols, and ultimately lead to safer and more effective surgical interventions.

## Figures and Tables

**Table 1:** Characteristics of the Studies.

Studies	Study Design	Discipline	Robotic Surgical System	Comparative Group
Kaouk et al., 2003 [7]	Case series	Urology	Da Vinci	no
van Mulken et al., 2020 [8]	RCT	Plastic Surgery	MUSA	yes
van Mulken et al., 2022 [9]	RCT	Plastic Surgery	MUSA	yes
Mori et al., 2024 [10]	Case series	Orthopedic Surgery	Symani	no
Struebing et al., 2024 [11]	Case series	Plastic Surgery	Symani	no
Wessel et al., 2024 [12]	Preclinical study	Plastic Surgery	Symani	no
Menichini et al., 2024 [13]	Preclinical study	Plastic Surgery	Symani	yes
Barbon et al., 2022 [14]	Case series	Plastic Surgery	Symani	yes
Lindenblatt et al., 2022 [15]	Case series	Plastic Surgery	Symani	no
Jiang et al., 2016 [16]	Preclinical study	Neurosurgery	Da Vinci	no
Malzone et al., 2023 [17]	Preclinical study	Plastic Surgery	Symani	yes
Reilly et al., 2024 [18]	Case series	Plastic Surgery	MUSA	no
Wessel et al., 2024 [19]	Preclinical study	Plastic Surgery	Symani	yes
Lilja et al., 2024 [20]	Case report	Plastic Surgery	Symani	no
Vollbach et al., 2024 [21]	Case report	Plastic Surgery	Symani	no
Tolksdorf et al., 2024 [22]	Case series	Maxillofacial surgery	Symani	yes
Innocenti et al., 2022 [23]	Case report	Plastic Surgery	Symani	no
Naito et al., 2020 [24]	Case report	Neurosurgery	Da Vinci	no
Besmens et al., 2024 [25]	Case series	Plastic Surgery	Symani	no
Lai et al., 2019 [26]	Case series	Maxillofacial surgery	Da Vinci	yes
Chang et al., 2021 [27]	Case report	Plastic Surgery	Da Vinci	no
Frieberg et al., 2024 [28]	Preclinical study	Maxillofacial surgery	MUSA	yes
Beier et al., 2023 [29]	Case series	Plastic Surgery	Symani	no
Ceccarelli et al., 2020 [30]	Case report	Vascular Surgery	Da Vinci	no
Gorji et al., 2024 [31]	Case series	Plastic Surgery	Symani	no
Rusch et al., 2024 [32]	Preclinical study	Cardiac Surgery	Symani	no
Grünherz et al., 2023 [33]	Case Report	Plastic Surgery	Symani	no
Dastagir et al., 2024 [34]	Case series	Plastic Surgery	Symani	yes
Chang et al., 2020 [35]	Case series	Thoracic Surgery	Da Vinci	no
Weinzierl et al., 2023 [36]	Case series	Plastic Surgery	Symani	no
von Reibnitz et al., 2024 [37]	Case series	Plastic Surgery	Symani	no
Nectoux et al., 2009 [38]	Preclinical study	Plastic Surgery	Da Vinci	no
Katz et al., 2005 [39]	Preclinical study	Maxillofacial Surgery	Da Vinci	yes
Miyamoto et al., 2014 [40]	Case series	Plastic Surgery	Da Vinci	no
Karamanoukian et al., 2006 [41]	Preclinical study	Plastic Surgery	ZEUS	yes
Arnold et al., 2002 [42]	Preclinical study	Cardiac Surgery	ZEUS	yes
Lee et al., 2012 [43]	Preclinical study	Plastic Surgery	Da Vinci	no
Latif et al., 2008 [44]	Preclinical study	Plastic Surgery	Da Vinci	no
Taleb et al., 2008 [45]	Preclinical study	Plastic Surgery	Da Vinci	no
Knight et al., 2005 [46]	Preclinical study	Plastic Surgery	ZEUS	yes
Ramdhian et al., 2011 [47]	Preclinical study	Plastic Surgery	Da Vinci	yes
Miyamoto et al., 2016 [48]	Preclinical study	Neurosurgery	Da Vinci	no
Karamanoukian et al., 2006 [49]	Preclinical study	Plastic Surgery	ZEUS	no
Katz et al., 2006 [50]	Preclinical study	Plastic Surgery	Da Vinci	no
Garcia et al., 2012 [51]	Case series	Plastic Surgery	Da Vinci	no
Facca et al., 2010 [52]	Case Report	Plastic Surgery	Da Vinci	no
Mantovani et al., 2011 [53]	Preclinical study	Neurosurgery	Da Vinci	no
Damiano et al., 2001 [54]	Clinical Trial	Cardiac Surgery	ZEUS	no

**Table 2:** Arterial anastomoses: robotic vs. manual.

Article	Robotic Anastomosis Time (min)	Manual Anastomosis Time (min)	Time Difference (min)	Difference Significance (*p*-Value)
Malzone et al., 2023 [17]	12.2	7.6	4.6	*p* < 0.0001
Tolksdorf et al., 2024 [22]	32.5	11.8	20.7	*p* < 0.001
Dastagir et al., 2024 [34]	17.3	16.1	1.2	*p* = 0.0883
Karamanoukian et al., 2006 [41]	14	7.2	6.8	*p* < 0.01
Knight et al., 2005 [46]	27.6	17.2	10.4	*p* = 0.0006

**Table 3:** Venous anastomoses: robotic vs. manual.

Article	Robotic Anastomosis Time (min)	Manual Anastomosis Time (min)	Time Difference (min)	Difference Significance (*p*-Value)
Malzone et al., 2023 [17]	19.6	13.2	6.4	*p* < 0.0001
Tolksdorf et al., 2024 [22]	41.7	13.4	28.3	*p* < 0.001
Dastagir et al., 2024 [34]	11.5	10.2	1.3	*p* = 0.0972

**Table 4:** Learning Curve in Anastomosis Time.

Studies	First Reported Anastomosis Time (min)	Last Reported Anastomosis Time (min)	Time Difference (min)	Improvement Rate (%)
van Mulken et al., 2020 [8]	33	16	17	51.5%
Struebing et al., 2024 [11]	n.s.	n.s.	n.s.	1.0%
Barbon et al., 2022 [14]	23.9	16.3	7	30.4%
Reilly et al., 2024 [18]	n.s.	n.s.	n.s.	30.0%
Wessel et al., 2024 [19]	29	19	10	34.5%
Frieberg et al., 2024 [28]	33.08	13.58	19.5	58.9%
Gorji et al., 2024 [31]	60	19	41	68.3%
Rusch et al., 2024 [32]	47.28	22.37	24.91	52.7%
Dastagir et al., 2024 [34]	21	14.7	6.3	30.0%
Weinzierl et al., 2023 [36]	59	20	39	66.10%
Lee et al., 2012 [43]	n.s.	n.s.	n.s.	n.s.
Knight et al., 2005 [46]	22.9	18.7	4.2	18.3%
Ramdhian et al., 2011 [47]	63.53	41.33	22.2	34.9%
Katz et al., 2006 [50]	70	20	50	71.4%
Garcia et al., 2012 [51]	n.s.	n.s.	n.s.	n.s.

## Data Availability

All data are available from the references cited in Table 1.

## Abbreviations

ICG	indocyanine green
LLAs	Lymphatic-lymphatic anastomoses
LVAs	Lymphatic-venous anastomoses
MeSH	Medical Subject Headings
Min	minutes
n.s.	not specified
PRISMA	Preferred Reporting Items for Systematic Reviews and Meta-Analyses
WoS	Web of Science
ZRSS	ZEUS Robotic Surgical System
RCT	Randomized controlled trial

## References

[1] Reddy K., Gharde P., Tayade H., Patil M., Reddy L.S., Surya D. (2023). Advancements in Robotic Surgery: A Comprehensive Overview of Current Utilizations and Upcoming Frontiers. Cureus.

[2] Yim N.H., Burns H.R., Davis M.J., Selber J.C. (2023). Robotic Plastic Surgery Education: Developing a Robotic Surgery Training Program Specific to Plastic Surgery Trainees. Semin. Plast. Surg..

[3] Rivero-Moreno Y., Echevarria S., Vidal-Valderrama C., Stefano-Pianetti L., Cordova-Guilarte J., Navarro-Gonzalez J., Acevedo-Rodríguez J., Dorado-Avila G., Osorio-Romero L., Chavez-Campos C. (2023). Robotic Surgery: A Comprehensive Review of the Literature and Current Trends. Cureus.

[4] Yohannes P., Rotariu P., Pinto P., Smith A.D., Lee B.R. (2002). Comparison of robotic versus laparoscopic skills: Is there a difference in the learning curve?. Urology.

[5] Leijte E., de Blaauw I., Van Workum F., Rosman C., Botden S. (2020). Robot assisted versus laparoscopic suturing learning curve in a simulated setting. Surg. Endosc..

[6] Page M.J., McKenzie J.E., Bossuyt P.M., McKenzie J.E., Bossuyt P.M., Boutron I., Hoffmann T.C., Mulrow C.D., Shamseer L., Tetzlaff J.M. (2021). The PRISMA 2020 statement: An updated guideline for reporting systematic reviews. BMJ.

[7] Kaouk J.H., Desai M.M., Abreu S.C., Papay F., Gill I.S. (2003). Robotic assisted laparoscopic sural nerve grafting during radical prostatectomy: Initial experience. J. Urol..

[8] van Mulken T.J.M., Schols R.M., Scharmga A.M.J., Winkens B., Cau R., Schoenmakers F.B.F., Qiu S.S., van der Hulst R.R.W.J., Keuter X.H.A., MicroSurgical Robot Research Group (2020). First-in-human robotic supermicrosurgery using a dedicated microsurgical robot for treating breast cancer-related lymphedema: A randomized pilot trial. Nat. Commun..

[9] van Mulken T.J.M., Wolfs J.A.G.N., Qiu S.S., Scharmga A.M.J., Schols R.M.M., van Weezelenburg M.A.S., Cau R., van der Hulst R.R.W.J.M., MicroSurgical Robot Research Group (2022). One-Year Outcomes of the First Human Trial on Robot-Assisted Lymphaticovenous Anastomosis for Breast Cancer-Related Lymphedema. Plast. Reconstr. Surg..

[10] Mori F., Menichini G., Rizzo F., Sassu P., Innocenti M. (2024). Robotic-Assisted Anastomosis in Orthoplastic Surgery: Preliminary Data. Handchir. Mikrochir. Plast. Chir..

[11] Struebing F., Bigdeli A., Weigel J., Gazyakan E., Vollbach F., Panayi A.C., Vogelpohl J., Boecker A., Kneser U., Struebing F. (2024). Robot-assisted Microsurgery: Lessons Learned from 50 Consecutive Cases. Plast. Reconstr. Surg. Glob. Open.

[12] Wessel K.J., Wendenburg I., Varnava C., Wellenbrock S., Dermietzel A., Hiort M., Kampshoff D., Wiebringhaus P., Hirsch T., Kueckelhaus M. (2024). Ideal suturing technique for robot-assisted microsurgical anastomoses. J. Robot. Surg..

[13] Menichini G., Malzone G., Tamburello S., Andreoli A.L., Mori F., Ballestín A., Shiraki T. (2024). Safety and efficacy of Symani robotic-assisted microsurgery: Assessment of vascular anastomosis patency, thrombus, and stenosis in a randomized preclinical study. J. Plast Reconstr. Aesthet. Surg..

[14] Barbon C., Grünherz L., Uyulmaz S., Giovanoli P., Lindenblatt N. (2022). Exploring the learning curve of a new robotic microsurgical system for microsurgery. JPRAS Open.

[15] Lindenblatt N., Grünherz L., Wang A., Gousopoulos E., Barbon C., Uyulmaz S., Giovanoli P. (2022). Early Experience Using a New Robotic Microsurgical System for Lymphatic Surgery. Plast. Reconstr. Surg. Glob. Open.

[16] Jiang S., Ichihara S., Prunières G., Peterson B., Facca S., Xu W.-D., Liverneaux P. (2016). Robot-assisted C7 nerve root transfer from the contralateral healthy side: A preliminary cadaver study. Hand Surg. Rehabil..

[17] Malzone G., Menichini G., Innocenti M., Ballestín A. (2023). Microsurgical robotic system enables the performance of microvascular anastomoses: A randomized in vivo preclinical trial. Sci. Rep..

[18] Reilly F.O.F., Nilsson A., Frieberg H., Mayr-Riedler M.S., Mani M. (2024). Implementation of robot-assisted lymphaticovenous anastomoses in a microsurgical unit. Eur. J. Plast. Surg..

[19] Wessel K.J., Stögner V.A., Yu C.T., Pomahac B., Hirsch T., Ayyala H.S., Kueckelhaus M.M. (2024). Preclinical Performance of the Combined Application of Two Robotic Systems in Microsurgery: A Two-center Study. Plast. Reconstr. Surg. Glob. Open.

[20] Lilja C., Thomsen J.B., Sørensen J.A. (2024). Robot-assisted lymphovenous anastomosis surgery for lymphocele in the groin. BMJ Case Rep..

[21] Vollbach F.H., Bigdeli A.K., Struebing F., Weigel J.L., Gazyakan E., Kneser U. (2024). Using a Microsurgical Robotic Platform for In-flap Anastomosis in Autologous Bipedicular Breast Reconstruction. Plast. Reconstr. Surg. Glob. Open.

[22] Tolksdorf K., Hohberger F.S., Ernst C., Tietz S., Schultze-Mosgau S., Tautenhahn F. (2024). First experience using a novel microsurgical robotic device for free flap surgery in cranio- and maxillofacial surgery. J. Craniomaxillofac. Surg..

[23] Innocenti M., Malzone G., Menichini G. (2023). First-in-Human Free Flap Tissue Reconstruction Using a Dedicated Microsurgical Robotic Platform. Plast. Reconstr. Surg..

[24] Naito K., Imashimizu K., Nagura N., Goto K., Obata H., Kaneko A., Sugiyama Y., Kaneko K. (2020). Robot-assisted Intercostal Nerve Harvesting: A Technical Note about the First Case in Japan. Plast. Reconstr. Surg. Glob. Open.

[25] Besmens I.S., Politikou O., Giovanoli P., Calcagni M., Lindenblatt N. (2024). Robotic Microsurgery in Extremity Reconstruction—Experience With a Novel Robotic System. Surg. Innov..

[26] Lai C.S., Lu C.T., Liu S.A., Tsai Y.C., Chen Y.W., Chen I.C. (2019). Robot-assisted microvascular anastomosis in head and neck free flap reconstruction: Preliminary experiences and results. Microsurgery.

[27] Chang T.N.J., Daniel B.W., Hsu A.T.W., Chen L.W., Sung C.W., Chuang D.C., Chao Y. (2021). Reversal of thoracic sympathectomy through robot-assisted microsurgical sympathetic trunk reconstruction with sural nerve graft and additional end-to-side coaptation of the intercostal nerves: A case report. Microsurgery.

[28] Frieberg H., Winter J.M., Engström O., Önefäldt D., Nilsson A., Mani M. (2024). Robot-Assisted Microsurgery-what does the learning curve look like?. JPRAS Open.

[29] Beier J.P., Hackenberg S., Boos A.M., Modabber A., Duong Dinh T.A., Hölzle F. (2023). First Series of Free Flap Reconstruction Using a Dedicated Robotic System in a Multidisciplinary Microsurgical Center. Plast. Reconstr. Surg. Glob. Open.

[30] Ceccarelli G., Gusai G., Rondelli F., Balestra F., De Rosa M. (2020). Video-robotic aneurysmectomy for splenic artery aneurysm: Case report and literature review. Minim. Invasive Ther. Allied Technol..

[31] Gorji S., Wessel K., Dermietzel A., Aitzetmueller M., Wendenburg I., Varnava C., Klietz M., Wiebringhaus P., Hirsch T., Kueckelhaus M. (2024). Fully Telemetric Robotic Microsurgery: Clinical Experience With 23 Cases. Microsurgery.

[32] Rusch M., Hoffmann G., Wieker H., Bürger M., Kapahnke S., Berndt R., Rusch R. (2024). Evaluation of the MMI Symani^®^ robotic microsurgical system for coronary-bypass anastomoses in a cadaveric porcine model. J. Robot. Surg..

[33] Grünherz L., Weinzierl A., Puippe G.D., von Reibnitz D., Barbon C., Schneider M.A., Giovanoli P., Gutschow C.A., Lindenblatt N. (2023). First-in-human Use of a Microsurgical Robotic System for Central Lymphatic Reconstruction. Plast. Reconstr. Surg. Glob. Open.

[34] Dastagir N., Obed D., Tamulevicius M., Dastagir K., Vogt P.M. (2024). The Use of the Symani Surgical System^®^ in Emergency Hand Trauma Care. Surg. Innov..

[35] Chang T.N.J., Chen L.W.Y., Lee C.P., Chang K.H., Chuang D.C.C., Chao Y.K. (2020). Microsurgical robotic suturing of sural nerve graft for sympathetic nerve reconstruction: A technical feasibility study. J. Thorac. Dis..

[36] Weinzierl A., Barbon C., Gousopoulos E., von Reibnitz D., Giovanoli P., Grünherz L., Lindenblatt N. (2023). Benefits of robotic-assisted lymphatic microsurgery in deep anatomical planes. JPRAS Open.

[37] von Reibnitz D., Weinzierl A., Barbon C., Gutschow C.A., Giovanoli P., Grünherz L., Lindenblatt N. (2024). 100 anastomoses: A two-year single-center experience with robotic-assisted micro- and supermicrosurgery for lymphatic reconstruction. J. Robot. Surg..

[38] Nectoux E., Taleb C., Liverneaux P. (2009). Nerve repair in telemicrosurgery: An experimental study. J. Reconstr. Microsurg..

[39] Katz R.D., Rosson G.D., Taylor J.A., Singh N.K. (2005). Robotics in microsurgery: Use of a surgical robot to perform a free flap in a pig. Microsurgery.

[40] Miyamoto H., Leechavengvongs S., Atik T., Facca S., Liverneaux P. (2014). Nerve transfer to the deltoid muscle using the nerve to the long head of the triceps with the da Vinci robot: Six cases. J. Reconstr. Microsurg..

[41] Karamanoukian R.L., Bui T., McConnell M.P., Evans G.R.D., Karamanoukian H.L. (2006). Transfer of training in robotic-assisted microvascular surgery. Ann. Plast. Surg..

[42] Arnold M., Boehm D.H., Welsch U., Detter C., Reichart B., Reichenspurner H. (2002). Evaluation of acute traumatic changes of the coronary artery wall after robotically assisted endoscopic coronary artery bypass grafting. Heart Surg. Forum.

[43] Lee J.Y., Mattar T., Parisi T.J., Carlsen B.T., Bishop A.T., Shin A.Y. (2012). Learning curve of robotic-assisted microvascular anastomosis in the rat. J. Reconstr. Microsurg..

[44] Latif M.J., Afthinos J.N., Connery C.P., Perin N., Bhora F.Y., Chwajol M., Todd G.J., Belsley S.J. (2008). Robotic intercostal nerve graft for reversal of thoracic sympathectomy: A large animal feasibility model. Int. J. Med. Robot..

[45] Taleb C., Nectoux E., Liverneaux P.A. (2008). Telemicrosurgery: A feasibility study in a rat model. Chir. Main..

[46] Knight C.G., Lorincz A., Cao A., Gidell K., Klein M.D., Langenburg S.E. (2005). Computer-assisted, robot-enhanced open microsurgery in an animal model. J. Laparoendosc. Adv. Surg. Tech. A.

[47] Ramdhian R.M., Bednar M., Mantovani G.R., Facca S.A., Liverneaux P.A. (2011). Microsurgery and telemicrosurgery training: A comparative study. J. Reconstr. Microsurg..

[48] Miyamoto H., Serradori T., Mikami Y., Selber J., Santelmo N., Facca S., Liverneaux P. (2016). Robotic intercostal nerve harvest: A feasibility study in a pig model. J. Neurosurg..

[49] Karamanoukian R.L., Finley D.S., Evans G.R.D., Karamanoukian H.L. (2006). Feasibility of robotic-assisted microvascular anastomoses in plastic surgery. J. Reconstr. Microsurg..

[50] Katz R.D., Taylor J.A., Rosson G.D., Brown P.R., Singh N.K. (2006). Robotics in plastic and reconstructive surgery: Use of a telemanipulator slave robot to perform microvascular anastomoses. J. Reconstr. Microsurg..

[51] Garcia J.C., Lebailly F., Mantovani G., Mendonca L.A., Garcia J., Liverneaux P. (2012). Telerobotic manipulation of the brachial plexus. J. Reconstr. Microsurg..

[52] Facca S., Liverneaux P. (2010). Robotic assisted microsurgery in hypothenar hammer syndrome: A case report. Comput. Aided Surg..

[53] Mantovani G., Liverneaux P., Garcia J.C., Berner S.H., Bednar M.S., Mohr C.J. (2011). Endoscopic exploration and repair of brachial plexus with telerobotic manipulation: A cadaver trial. J. Neurosurg..

[54] Damiano R.J., Tabaie H.A., Mack M.J., Edgerton J.R., Mullangi C., Graper W., Prasad S.M. (2001). Initial prospective multicenter clinical trial of robotically-assisted coronary artery bypass grafting. Ann. Thorac. Surg..

[55] Sadri H., Fung-Kee-Fung M., Shayegan B., Garneau P.Y., Pezeshki P. (2023). A systematic review of full economic evaluations of robotic-assisted surgery in thoracic and abdominopelvic procedures. J. Robot. Surg..

[56] Childers C.P., Maggard-Gibbons M. (2018). Estimation of the Acquisition and Operating Costs for Robotic Surgery. JAMA.

